# Preparation of Room Temperature Vulcanized Silicone Rubber Foam with Excellent Flame Retardancy

**DOI:** 10.1155/2021/9976005

**Published:** 2021-05-20

**Authors:** Weiqi Luo, Zhimin Li, Haihua Luo, Yuting Liu, Guojiang Xia, Hangtian Zhu, Jiayi Zhou, Ding Yu, Jianxin Zhang, Jianghang Song, Zhengzhou Duan, Yanxin Qiao, Jijun Tang, Yuxin Wang, Chunfeng Meng

**Affiliations:** School of Material Science and Engineering, Jiangsu University of Science and Technology, Zhenjiang, Jiangsu 212003, China

## Abstract

To retard the spread of fire in many cases with sealing materials is significant. A series of silicone rubber foam materials were prepared with room temperature vulcanization and foaming reactions. The morphology, chemical structure, cell structure, and thermal stability were investigated and results proved that the synthesis of silicone rubber was successful in a wide range of feed ratios. The fire-retardant tests were carried out to study the fire-proof property of the composite materials, and the excellent performance showed a promising prospect for wide application in sealing materials.

## 1. Introduction

Large-scale flames can spread through the holes and gaps around the pipes, and cable trenches, when an accidental fire occurs. The runoff of fire can lead to a devastating outcome. According to the data released by the Emergency Management Department Fire Rescue Bureau of China, a total of 196,000 fire accidents were reported from January to October, in the year 2020, causing 889 deaths, 583 injuries, and direct property losses up to 2.55 billion yuan. The chance of tragedies can be minimized only with particular precaution measures. Herein, to retard the spread of fire in buildings with sealing materials is significant and life-saving.

In the field of fire-retardant materials, industrialization production is busy due to the market demand. A series of polymer-based composite materials, such as styrene butadiene rubber [1–3], natural rubber [4, 5], and so on are often used as fire-proof sealing materials to retard flames [6], especially in nuclear power stations, railway traffic, through-the-wall cable channels, and other fields. Generally, the composite materials are categorized into two types according to the relation between the retardant and the polymer material, which are reaction-type and additive-type [7]. In recent years, silicone rubber foam has been one of the most successful commercialized additive-type fire-proof products [8, 9], due to the comparatively higher thermal stability of silicones than their polymer counterparts, the shielding effect provided by the residue silica ash formed in pyrolysis, excellent aging-resistant performance of polysilicone, outstanding smoke suppression ability, low mass density, nontoxicity, electrical insulating property, and so on [9]. Though silicone rubbers combust lower heat than other polymers, they are still flammable and ignitable. Therefore, inorganic flame retardants, such as Al(OH)_3_, red phosphorus, Fe_2_O_3_, and SiO_2_, are usually incorporated into silicone rubbers to fabricate fire-proof products [10, 11].

However, the synergetic effect between the polymer matrix and inorganic fire-retardant and the total performance of the composites are sometimes too intricate to evaluate. For example, some inorganic flame retardant additives themselves are controversial enough as the released toxic gas and smoke are extremely harmful despite the outstanding flame-retardant property [12]. Besides, the content of the inorganic filler as a function of fire retardant is limited by the chemical and physical property of the rubber matrix. Therefore, silane-modified polyether can be added to the rubber matrix, as the low viscosity makes it possible to add more flame retardant additives.

The solidification reaction is difficult to control when non-reactive components are doped in the material. Herein, in this work, we presented the synthesis of fire-retardant room-temperature-vulcanized silicone rubber foam by a facial way, and aluminum hydroxide was incorporated as a flame retardant. The experiments were carried in a wide range of feed ratios, and the flame retardancy, compression stress-strain properties of the silicone rubber foams were systematically studied. These nontoxic, smokeless, and halogen-free silicone rubber foams can be applied as excellent fire-proof sealing materials.

## 2. Experimental

### 2.1. Materials

*α*, *ω*-dihydroxypolydimethylsiloxane (HO-PDMS-OH, purity ≥98%, viscosity 750 mPa s, Shandong Dayi Chemical Co., Ltd.), divinylpolydimethylsiloxane (Vi-PDMS-Vi, purity ≥99%, viscosity 3500 mPa S, vinyl content wt% 0.43, Shandong Dayi Chemical Co., Ltd.), polymethylvinylsiloxane (PMVS, purity ≥99%, viscosity 3500 mPa s, vinyl content wt% 0.7, Shandong Dayi Chemical Co., Ltd.), polymethylhydrosiloxane (PMHS, purity ≥98%, viscosity 20 mPa s, active hydrogen content wt% ≥1.5, Shandong Dayi Chemical Co., Ltd.), and Silane modified polyether (SMP, viscosity 36000-42000 mPa s, Risun Polymer) were purchased and used directly. Aluminum hydroxide (Al(OH)_3_, Zhengzhou Beifang Aluminum Co., Ltd.) Silica (Wacker International Group Co., Ltd), platinum catalyst (Shandong Dayi Chemical Co., Ltd), and carbon black (Anyang Tongsheng Carbon Black Co., Ltd.) were used as additives.

### 2.2. Preparation of Silicone Foam Materials

**Figure 1:**
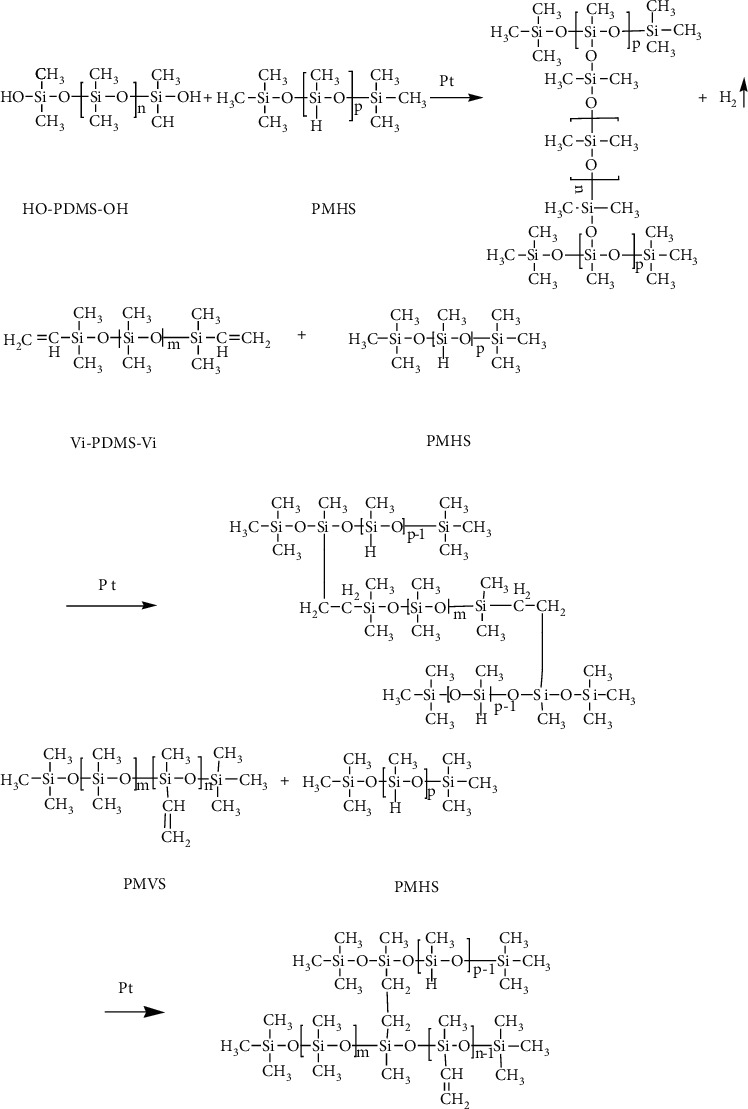
The foaming (up) and vulcanization reactions (middle and down).

Vi-PDMS-Vi and PMVS with a weight ratio of 1 : 1 were mixed first, and then, HO-PDMS-OH, SMP, PMHS, catalyst, polymerization inhibitor, and other dopants were added and stirred to form a homogenous mixture, with weight ratios shown in Table 1. The viscous liquids were kept still at room temperature in a proper mold until complete vulcanization was reached, and silicone foam materials were obtained. The vulcanization and foaming reactions are shown in Figure 1.

### 2.3. Characterization

The apparent densities of the silicone rubber were calculated by Equation (1), referring to ISO 845-2006:

(1) $$\begin{array}{c} \rho =\frac{m}{V}\times 10^{6}, \end{array}$$

where *m* is the mass of the sample in grams, *V* is the volume of the sample in cubic millimeters. Each sample was cut to a cubic with a size of 100 mm × 100 mm × 100 mm, and the apparent density of each sample was decided by the average value of five specimens. The expansion ratios were calculated by the volume ratios of the silicone rubbers after and before the vulcanization reaction, according to Equation (2)

(2) $$\begin{array}{c} \frac{V_{\text{g}}-V_{\text{i}}}{V_{\text{g}}}\times 100, \end{array}$$

where *V*_*g*_ is the geometrical volume of the test specimen, 10 mm × 10 mm × 10 mm, and *V*_*i*_ is the volume of the tested specimen into which water cannot get in or out. To measure *V*_*i*_, a certain amount of water was put into a graduated cylinder and recorded as *V*_1_. Then, the specimen with a size of 10 mm × 10 mm × 10 mm was put immersed, and the total volume was recorded as *V*_2_, so the *V*_*i*_ = *V*_2_ − *V*_1_. The microstructure was studied with a field-emission scanning electron microscopy (ZEISS Merlin Compact).

The cell densities of the samples were tested with the following equation, referring to the literature [13, 14].

(3) $$\begin{array}{c} N_{0}={\left[\frac{{nM}^{2}}{A}\right]}^{\frac{3}{2}}\varphi , \end{array}$$

where *n*, *M*, *A*, and *ϕ* represented the pore number in SEM image, the amplification factor, the area of SEM image, and the expansion ratio. The chemical structure of the silicone foams was investigated with ATR-FTIR, with a Nicolet IS 10 equipment in a wavenumber range of 4000-500 cm^−1^. The thermal stability of silicone foams was studied with a thermogravimetric analysis device (PerkinElmer, Pyris Diamond TG-DTA), from room temperature to 900°C, at a ramp rate of 10°C min^−1^, in the air. The stress-strain curves were measured with a universal testing machine (WAW/WEW-1000D, Jinanshidai Testing Machine Co., Ltd.) and recorded according to GB/T 8168-2008. The size of the specimen was 100 mm × 100 mm × 25 mm, and three specimens were tested for each sample with a preload of 2 N. The load of the compressing plate to the specimen was gradually increased along the thickness direction at a rate of 12 mm min^−1^ until the specimen was completely broken. The compressive strain was calculated by Equation (4)

(4) $$\begin{array}{c} \varepsilon_{\alpha }=\frac{T-T_{\text{i}}}{T}, \end{array}$$

where *ε*_*α*_ is the compression deformation (%), *T* is the original height before compression, and *T*_i_ is the height after compression. And the compressive stress was calculated following the formula

(5) $$\begin{array}{c} \sigma =\frac{P}{A}\times 10^{-6}, \end{array}$$

where *σ* is the press stress (Pa), *P* is the loaded force (N), and *A* is the base area of the specimen (mm^−2^). The fire-retardancy property of the composites was studied with the vertical burning tests according to the UL-94 method [15, 16], and the materials are classified as V0, V1, or V2. The specimens with a size of 125 mm × 13 mm × 1.6 mm were ignited with methane. 10 seconds later, the methane was removed and the first afterflame time for the specimen was recorded as *t*_1_. Once the first afterflame is extinguished, the specimen was ignited again for another 10 seconds, and the second afterflame time (*t*_2_) and afterglow time (*t*_3_) were noted. A piece of cotton was placed under the specimen, and if the cotton was ignited by the burning drops, the results should be recorded.

## 3. Results and Discussion

### 3.1. Preparation of the Composite Materials

Samples were synthesized with vulcanization and foaming reactions at room temperature. As shown in Figure 1, vulcanization reactions between Vi-PDMS-Vi, PMVS, and PMHS cross-linked to form the skeleton of the composite material, and the reaction between HO-PDMS-OH and PMHS caused the foaming structure. After 72 hours, all the samples were solidified.

**Figure 2:**
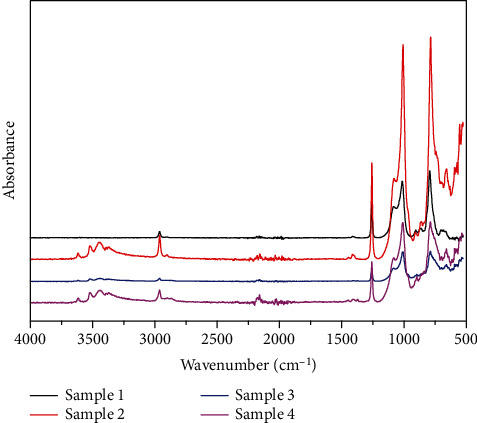
The ATR-FTIR spectra of the composite materials.

ATR-FTIR was carried out to study the chemical structures of the samples, and the results were shown in Figure 2. The peak at 3620 cm^−1^ was the signal of Si-OH bond of residual HO-PDMS-OH, and the multiple peaks near 3500 cm^−1^ were the signals of Si-OH bonds. In all four samples, Si-CH = CH_2_ bond around 1620 cm^−1^ was not detected, signifying the reactions were completed. The weak peak at 2150 cm^−1^ was the characteristic signal of Si-H, declaring the complete consumption of PMHS. The degree of vulcanization reaction affected the curing parameters of the rubber foam and the mechanical strength of the matrix. The strong peak at 1250 cm^−1^ was caused by the Si- CH_3_, and the Si-O-C peaks appeared in the range of 1000 to 1080 cm^−1^ [17]. The ATR-FTIR results proved the samples were successfully synthesized.

**Figure 3:**
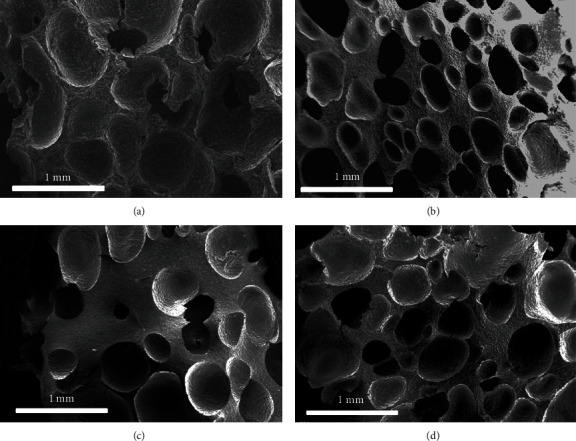
The SEM pictures of the composite materials. (a–d) Corresponding to Sample 1-4.

The morphology of the prepared composite materials was recorded with SEM, as shown in Figure 3. The cell diameters of the four composite materials, formed in the foaming process, were close and in a range of 0.1-0.8 mm. The contained additives were dispersed uniformly in the silicone rubber and caused the roughness of the cell wall. The cell structure ensured the silicone rubber high expansion ratios after vulcanization, as well as good stress-strain capacity, which was good for effective sealing.

### 3.2. Thermal Stability of the Composite Materials

**Figure 4:**
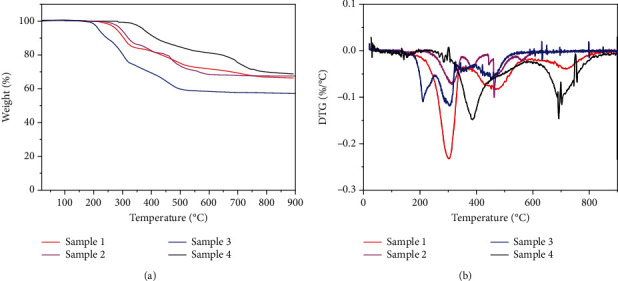
The thermogravimetry of the composite materials.

The thermal stability of fire-proof sealing materials is essential, and thermogravimetry was applied to investigate the four samples, as shown in Figures 4(a) and (b). All samples experienced multistep decomposition reactions. The decomposition process of Sample 1 started from 280°C, where the residual weight percent of Sample 1 was 95%. The thermal stability of Sample 2 was close to Sample 1. The thermal stability of sample 3 was relatively poorer than the other three analogs, as the decomposition process started from 200°C. The first degradation reaction of Sample 4 with a higher amount of Al(OH)_3_ started from 380°C, which was 100°C higher than Sample 1. It can be found from the related TGA data (Figure 4(b)) that the peak temperatures of the first pyrolysis were 303 and 388°C for Sample 1 and Sample 4, respectively. The addition of Al(OH)_3_ increased the thermal stability of the silicone rubber, which was helpful for fire-retardant materials. After decomposition, only SiO_2_ and Al_2_O_3_ remained, and the left weight percentages of Sample 1-4 were 66%, 67%, 57%, and 69%, respectively. The left nonflammable SiO_2_ and Al_2_O_3_ can act as shielding to stop the fire from spreading, which was the advantage of silicone rubber-based fire-retardant composite materials. The residue increment of Sample 2-4 comparing to Sample 1 was caused by the added amount of inorganic component Al(OH)_3_.

### 3.3. Mechanical Properties of the Composite Materials

The mechanical properties of the composite materials are crucial to the application of fire-retardant materials, and the apparent densities, expansion ratios, and porosities of the composite materials are shown in Table 2. The values of apparent densities ranged from 0.456 to 0.483 g cm^−3^, and the discrepancy was inconspicuous. The measured expansion ratios were between 2.73 and 2.79, endowing the composites as sealing materials for the holes and cracks. The calculated cell densities of the four samples were 1.7 × 10^9^, 2.9 × 10^9^, 1.2 × 10^9^, and 1.9 × 10^9^ pores per cm^3^. The size of pores was not evenly distributed. These data indicated that the silicone rubber foam can be successfully synthesized within a wide range of feed ratios.

**Figure 5:**
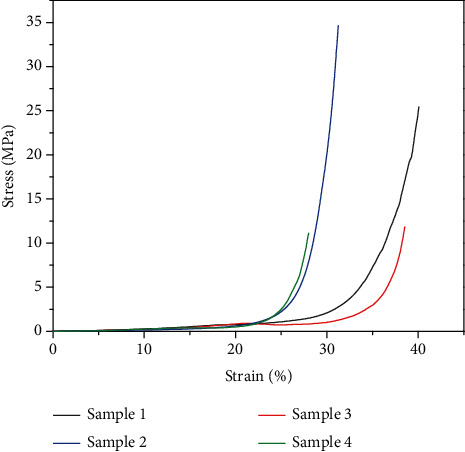
Stress-strain curves of the composite materials.

The stress-strain curves of the composite materials were recorded with a universal testing machine, as presented in Figure 5. The results were consistent with the mode of conventional foams proposed by Gibson and Ashby [18, 19]. As the strain increased from zero to a certain value, the stress-strain relationships were approximately linear, and the rising trends of stress were gradual and smooth. The flat trends can be caused by the bending of cell walls or the minimum collapse of foam structure, corresponding to the compressive elastic region. Then, the stress increased sharply with the strain, due to the massive collapse of the cell structure. For Sample 1-4, the starting strains where the foam structure began to collapse were 28%, 23%, 32%, and 22%, respectively. The four samples displayed good endurance to the stress, which is useful since the foaming materials should resist the consequential mechanical stress of the heat runaway and blasts of airflow.

**Figure 6:**
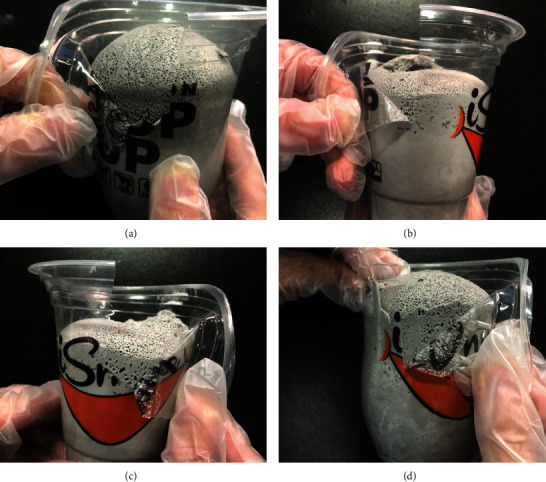
Optical images of the composite materials.

When the foaming composites are used as fireproof sealing materials, their adhesive performance to the surface of other materials is also essential, especially for the plastic materials. However, the surface energy of routine silicone rubber is low, so generally, the adhesion of routine silicone rubber is poor. In practical application, the sealing materials should adhere to the walls and gaps of plastic or metal pipes as long as possible. The adhesive performance of the samples to the surface of the commercial plastic cups is shown in Figure 6. After pealling off, the attachment remained on the inner walls of plastic walls, declaring the stickness of the composites to the plastic materials. Therefore, the samples are applicable for fireproof sealing materials.

### 3.4. Fire-Retardant Properties of the Composite Materials

The UL-94 tests of the composite materials are listed in Table 3. Firstly, the afterflame time (*t*_1_ or *t*_2_) for each individual specimen was less than 10 seconds. Secondly, the total afterflame time (*t*_1_ + *t*_2_) for any condition set for each specimen was less than 50 s. Thirdly, the afterflame plus afterglow time for each specimen after the second flame application was less than 30 s. Meanwhile, afterflame or afterglow of any specimen up to the holding clamp was not observed, nor the cotton indicator was ignited by flaming particles or drops. Therefore, all samples were rating in V0 [15, 16, 20]. The excellent fire-retardant properties were endowed by the synergetic effect of silicone rubber and the fire-retardant additives and ensured the composite materials practical application foreground in fireproof sealing.

## 4. Conclusion

In this work, a series of silicone rubber foam was prepared through room temperature vulcanization and foaming. The characterization results proved that the composite materials had been successfully synthesized in a wide range of feed ratios, and the cell structures and other mechanical properties were suitable for fire-retarding application. The composite materials exhibited excellent thermal stability. Fire-retardant tests proved the outstanding fireproof property for all four samples. In summary, the composite materials displayed good overall performance as fire-retardant materials.

## Figures and Tables

**Table 1:** Composition of raw materials (unit: g).

Sample	Vi-PDMS-Vi	PMVS	SMP	HO-PDMS-OH	Al(OH)3	Silica	Carbon black	Polymerization inhibitor	PMHS	Pt-catalyst
1	15	15	20	70	80	2	0.5	0.9	45	0.5
2	12.5	12.5	25	60	90	3	0.4	0.7	40	0.5
3	10	10	30	50	110	4	0.2	0.5	30	0.5
4	5	5	40	40	120	5	0.2	0.5	45	0.5

**Table 2:** Properties of the prepared composite materials.

Sample	1	2	3	4
Apparent density (g cm^−3^)	0.473	0.456	0.483	0.465
Expansion ratio	2.73	2.76	2.79	2.77
Porosity (%)	56.34	57.62	56.73	55.78

**Table 3:** Flame retardant test results.

	Specimen	t_1_	t_2_	t_3_	t_1_ + t_2_	t_2_ + t_3_	Afterflame or afterglow to the holding clamp	Cotton ignited by burning drops	UL-94 rate
Sample 1	1	5	4	15	9	19	No	No	V0
	2	5	5	14	10	19	No	No	
	3	6	4	15	10	19	No	No	
	4	5	3	14	8	17	No	No	
	5	6	5	14	11	19	No	No	

Sample 2	1	4	5	14	9	19	No	No	V0
	2	5	5	14	10	19	No	No	
	3	5	4	13	9	17	No	No	
	4	4	4	13	8	17	No	No	
	5	5	4	13	9	17	No	No	

Sample 3	1	4	4	13	8	17	No	No	V0
	2	5	4	13	9	17	No	No	
	3	4	4	12	8	16	No	No	
	4	4	4	13	8	17	No	No	
	5	4	5	11	9	16	No	No	

Sample 4	1	3	3	12	6	15	No	No	V0
	2	3	4	12	7	16	No	No	
	3	4	4	11	8	15	No	No	
	4	4	3	12	7	15	No	No	
	5	3	4	11	7	15	No	No	

## Data Availability

All data and models generated or used during the study appear in the submitted article.

## References

[1] Imiela M., Anyszka R., Bieliński D. M., Lipińska M., Rybiński P., Syrek B. (2019). Synergistic effect of mica, glass frit, and melamine cyanurate for improving fire resistance of styrene-butadiene rubber composites destined for ceramizable coatings. *Coatings*.

[2] Attia N. F., Saleh B. K. (2020). Novel synthesis of renewable and green flame-retardant, antibacterial and reinforcement material for styrene–butadiene rubber nanocomposites. *Journal of Thermal Analysis and Calorimetry*.

[3] Abdel-Hakim A., El-Basheer T. M., Abdelkhalik A. (2020). Mechanical, acoustical and flammability properties of SBR and SBR-PU foam layered structure. *Polymer Testing*.

[4] Intharapat P., Nakason C., Kongnoo A. (2016). Preparation of boric acid supported natural rubber as a reactive flame retardant and its properties. *Polymer Degradation and Stability*.

[5] Wang N., Xu G., Wu Y. (2016). The influence of expandable graphite on double-layered microcapsules in intumescent flame-retardant natural rubber composites. *Journal of Thermal Analysis and Calorimetry*.

[6] Sun Z. E., Zhou Y. (2016). Discussion on fire-proof sealing technology and product. *Procedia Engineering*.

[7] Camino G., Costa L., di Cortemiglia M. P. L. (1991). Overview of fire retardant mechanisms. *Polymer Degradation and Stability*.

[8] Zhu C., Deng C., Cao J. Y., Wang Y. Z. (2015). An efficient flame retardant for silicone rubber: preparation and application. *Polymer Degradation and Stability*.

[9] Lou F., Yan W., Guo W., Wei T., Li Q. (2017). Preparation and properties of ceramifiable flame-retarded silicone rubber composites. *Journal of Thermal Analysis and Calorimetry*.

[10] Shah A. U. R., Prabhakar M. N., Song J.-I. (2017). Current advances in the fire retardancy of natural fiber and bio-based composites – a review. *International Journal of Precision Engineering and Manufacturing-Green Technology*.

[11] Gallo E., Schartel B., Braun U., Russo P., Acierno D. (2011). Fire retardant synergisms between nanometric Fe2O3 and aluminum phosphinate in poly(butylene terephthalate). *Polymers for Advanced Technologies*.

[12] Shi Y., Yu B., Zheng Y. (2018). A combination of POSS and polyphosphazene for reducing fire hazards of epoxy resin. *Polymers for Advanced Technologies*.

[13] Yin D., Mi J., Zhou H., Wang X., Tian H. (2020). Fabrication of branching poly (butylene succinate)/cellulose nanocrystal foams with exceptional thermal insulation. *Carbohydrate Polymers*.

[14] Li Y., Yin D., Liu W., Zhou H., Zhang Y., Wang X. (2020). Fabrication of biodegradable poly (lactic acid)/carbon nanotube nanocomposite foams: significant improvement on rheological property and foamability. *International Journal of Biological Macromolecules*.

[15] de Freitas Rocha M. A., Landesmann A., da Silva Ribeiro S. P., Martins R. C. (2019). Enhancement of fire retardancy properties of glass fibre–reinforced polyesters composites. *Fire and Materials*.

[16] (2013). *UL 94–Standard for Tests for Flammability of Plastic Materials for Parts in Devices and Appliances*.

[17] Tan Y., Yao J., Zhu H. (2020). Preparation of room temperature vulcanized silicone rubber foam/SiO2 nanocomposite and its fatigue buffering performance. *Journal of Macromolecular Science, Part A Pure and Applied Chemistry*.

[18] Xiang B., Jia Y., Lei Y. (2019). Mechanical properties of microcellular and nanocellular silicone rubber foams obtained by supercritical carbon dioxide. *Polymer Journal*.

[19] Gibson L. J., Ashby M. F. (1997). *Cellular Solids: Structure and Properties*.

[20] Chen W., Xu C., Liu Y., Liu Y., Wang Q. (2017). Synthesis and properties of an intrinsic flame retardant silicone rubber containing phosphaphenanthrene structure. *RSC Advances*.

